# Imlunestrant a next-generation oral SERD overcomes *ESR1* mutant resistance in estrogen receptor–positive breast cancer

**DOI:** 10.1172/jci.insight.188051

**Published:** 2025-05-06

**Authors:** Shira Sherman, Zachary M. Sandusky, Douglas Russo, David Zak, Agostina Nardone, Delia Friel, Francisco Hermida-Prado, Capucine Heraud, Genevra Kuziel, Ana Verma, Giorgio Gaglia, Sheheryar Kabraji, Quang-De Nguyen, Sandro Santagata, Sean W. Fanning, Rinath Jeselsohn

**Affiliations:** 1 Department of Medical Oncology and; 2 Center for Functional Cancer Epigenetics, Dana-Farber Cancer Institute, Boston, Massachusetts, USA.; 3 Harvard Medical School, Boston, Massachusetts, USA.; 4 Department of Data Science, Dana-Farber Cancer Institute, Boston, Massachusetts, USA.; 5 Department of Cancer Biology, Loyola University Stritch School of Medicine, Maywood, Illinois, USA.; 6 Ludwig Center at Harvard and; 7 Department of Pathology, Brigham and Women’s Hospital, Harvard Medical School, Boston, Massachusetts, USA.; 8 Susan F. Smith Center for Women’s Cancers and; 9 Lurie Family Imaging Center, Center for Biomedical Imaging in Oncology, Dana-Farber Cancer Institute, Boston, Massachusetts, USA.

**Keywords:** Cell biology, Oncology, Breast cancer

## Abstract

Estrogen receptor α (ER) is a critical driver of tumorigenesis and tumor progression in most breast cancers. Endocrine therapies (ET) targeting ER are central to treating hormone receptor–positive breast cancer, but resistance poses a clinical challenge. Some resistance mechanisms, particularly those involving estrogen-independent activity such as the *ESR1* mutations, rely on ER signaling, supporting the need for next-generation ET. We investigated the preclinical efficacy of imlunestrant, an oral selective ER degrader, in ER-positive breast cancer preclinical models, including models harboring the Y537S *ESR1* mutation, an activating mutation. Imlunestrant demonstrated antagonistic activity and effective degradation of both WT and mutant ER, resulting in cell growth suppression. In vivo, imlunestrant outperformed fulvestrant, leading to tumor regression in a patient-derived xenograft harboring the Y537S *ESR1* mutation. Cyclic mutiplexed immunofluorescence and transcriptomic analysis revealed enhanced cell cycle arrest and downregulation of estrogen-responsive genes with imlunestrant treatment. Additionally, a genome-wide CRISPR knock–out screen identified several vulnerabilities that were either persistent or acquired after imlunestrant treatment, providing a rationale for future studies of combination treatments with imlunestrant. Collectively, these results highlight the on-target and selective activity of imlunestrant, which can circumvent resistance engendered by the Y537S *ESR1* mutation.

## Abstract 2

Imlunestrant, a next-generation oral selective estrogen receptor degrader effectively suppresses ER-positive breast cancer, including resistant Y537S ESR1 mutations, supporting its clinical development for improved outcomes

## Introduction

Estrogen receptor α (ER) is a nuclear hormone receptor and a key driver of tumorigenesis and tumor progression in most breast cancers. Estrogen binding to ER leads to its activation, a process that involves ER dimerization, phosphorylation, and translocation to the nucleus where ER binds to cofactors and chromatin at estrogen response element (ERE) sites and directly mediates the transcription of hundreds of genes. The transcriptional activity of ER is dictated by other transcription factors, such as the pioneer transcription factor FOXA1, and is tightly regulated by coregulators that either activate or repress ER activity (1, 2).

Endocrine therapy that targets ER is the mainstay treatment in hormone receptor–positive (HR^+^) breast cancers, which express ER and/or the progesterone receptor (PR). There are several classes of endocrine therapies, including selective estrogen receptor modulators (SERMs), such as tamoxifen; aromatase inhibitors that suppress the conversion of androgens to estrogen and production of estrogen from peripheral tissue; selective estrogen receptor degraders (SERD) that are pure ER antagonists and enhance the proteasomal degradation of ER; and suppression of ovarian estrogen production in premenopausal women with GNRH analogs or bilateral salpingo-oophorectomy. Currently, the only SERD that is approved for all patients with metastatic HR^+^ breast cancer is fulvestrant. Although endocrine therapy has a substantial impact on outcomes in early and advanced stages of HR^+^ breast cancer, endocrine resistance is a major clinical obstacle. Multiple mechanisms of endocrine resistance exist, and several are unique to a specific class of endocrine therapy (3). As an example, the agonistic activity that drives resistance is unique to tamoxifen, and, in the clinic, is evidenced by the tumor response detected after tamoxifen withdrawal (4).

Estrogen independent activity of ER is a mechanism of resistance that is more specific to aromatase inhibitors. Several studies have shown that the crosstalk between ER and receptor tyrosine kinase signaling followed by activation of the PI3Kinase-AKT-MTOR and MAPK pathways results in estrogen-independent phosphorylation and activation of ER (5, 6). *ESR1* ligand binding missense mutations are predominantly acquired during treatment with aromatase inhibitors in metastatic disease, and, after such treatment in metastatic disease, these alterations are detected in more than 50% of patients when testing circulating tumor DNA (ctDNA) (7). These mutations, especially Y537S, reduce binding affinities of SERMs and SERDs while also impairing their abilities to engage the therapeutic conformation of the ligand binding domain (8–10). Overall, in preclinical studies the Y537S *ESR1* mutation was found to possess the most potent resistance to ER antagonists and ligand independent activity (11). Clinical observations further support the relative resistance to fulvestrant in the presence of a Y537S *ESR1* mutation (12), which is likely attributed to the poor bioavailability of this drug coupled with the increased quantities required to saturate the mutant receptor (10).

Preclinical studies have shown that *ESR1* mutations have neomorphic properties that promote a metastatic phenotype, and targeting these mutations leads to metastatic regression (13). Thus, *ESR1* mutations are a biomarker of an active but altered ER axis and a therapeutic target. Taken together, the high prevalence of these mutations and their biological implications warrant the development of novel endocrine therapies that have superior pharmacokinetics and/or better affinity mutant ER capable of destabilizing WT and mutant ER. Recently, elacestrant, a next-generation endocrine therapy with SERD activity, was approved for the treatment of *ESR1* mutant metastatic breast cancer (14). Additionally, there are several other novel endocrine therapies in clinical development (15). Imlunestrant is a novel SERD in clinical development with an excellent safety profile and a strong signal of activity in metastatic HR^+^ breast cancer (16, 17). In this study, we investigated the preclinical anticancer activities of imlunestrant with an emphasis on its effectiveness in the presence of the Y537S *ESR1* mutation.

### Imlunestrant has pure antagonistic activity and is a potent degrader of mutant ER.

**Figure 1:**
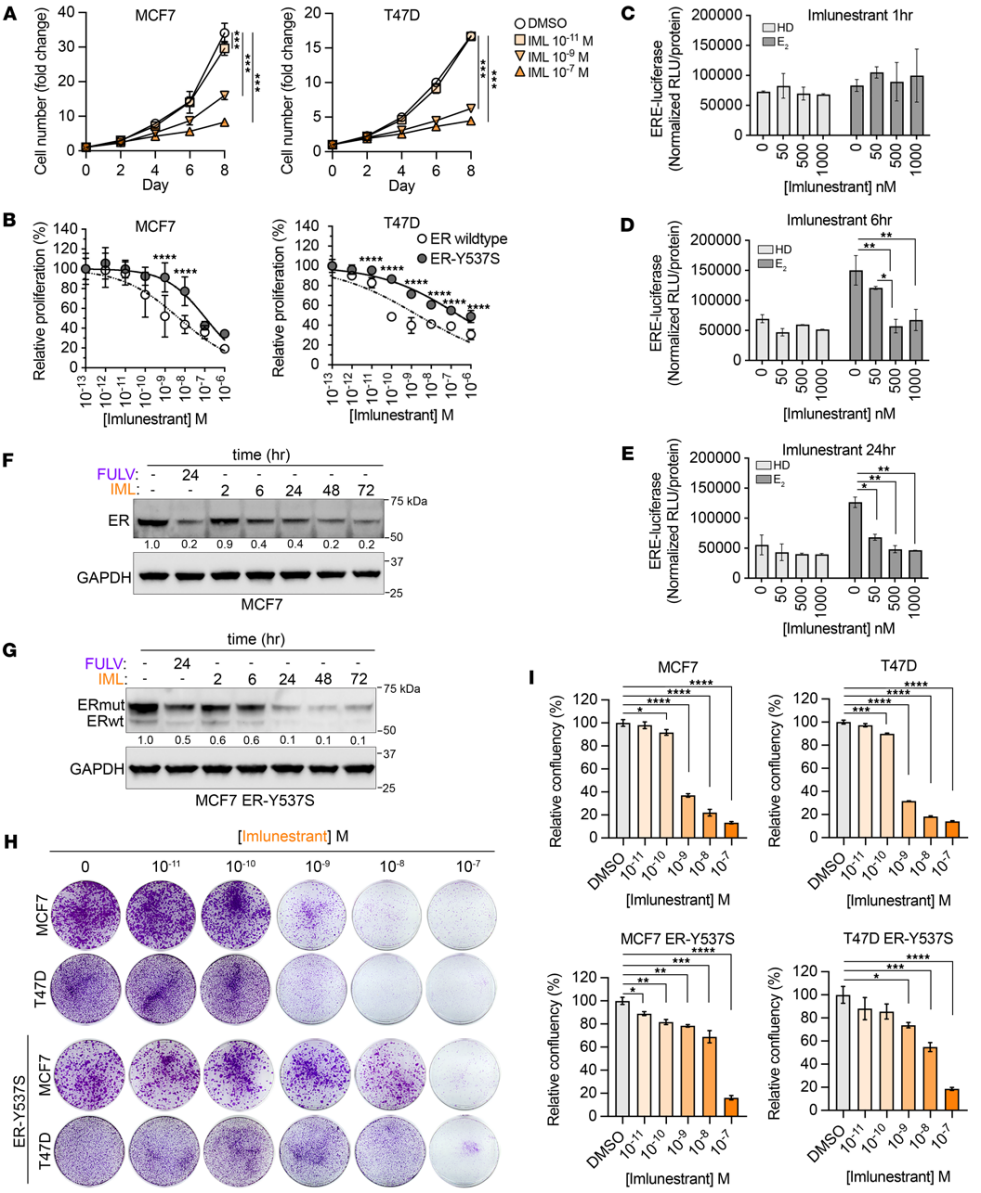
Antagonistic and ER degradation activity of imlunestrant. (**A**) Cell growth studies depicted as cell number fold change for MCF7 (left) or T47D (right) cells during treatment up to 8 days with DMSO or imlunestrant (IML). Error bars denote ± SD. Two-way ANOVA with Dunnett’s multiple comparisons test. (**B**) Normalized cell proliferation for MCF7 (left) or T47D (right) cells expressing ER-WT or ER-Y537S and treated with a dose-response of imlunestrant for 5 days. Error bars with average ± SD. Two-way ANOVA with Šidák’s multiple comparisons test. (**C**) Normalized luciferase signal in MCF7 ERE-LUC cells after hormone deprivation (HD) and treatment with or without E_2_ (1 nM) and imlunestrant (0, 50 nM, 500 nM, or 1,000 nM) for 1 hour, (**D**) 6 hours, or (**E**) 24 hours. Bar graph with averages. Error bars denote ± SD. Two-way ANOVA with Tukey’s multiple comparisons test. (**F**) Western blot of whole cell lysates for ER and GAPDH in MCF7 cells in HD media treated with fulvestrant (FULV; 100 nM, 24 hours) or imlunestrant (IML; 100 nM from 0–72 hours), as indicated. Densitometry of ER levels normalized to DMSO and GAPDH. (**G**) Western blot for ER WT (lower band) and ER mutant (HA-tagged, upper band) and GAPDH in MCF7 ER-Y537S cells in HD media treated with FULV (100 nM, 24 hours) or IML (100 nM, 0 to 72 hours), as indicated. Densitometry of ER mutant levels normalized to DMSO and GAPDH. (**H**) Colony assay crystal violet staining results from MCF7 or T47D cells with ER WT or ER-Y537S expression and treatment with imlunestrant (0 to 100 nM, as indicated) for 2 weeks in full media. (**I**) Relative confluency of colony assay crystal violet staining in **H**. Bar graph with average ± SD. One-way ANOVA with Dunnett’s multiple comparisons test. *P < 0.05, **P < 0.01, ***P < 0.001, ****P < 0.0001.

To characterize the preclinical activity of imlunestrant, we used MCF7 and T47D ER^+^ breast cancer model cell lines without and with doxycycline (DOX) induction of the Y537S *ESR1* mutation (13). We first evaluated the activity of imlunestrant on cell growth and observed dose-dependent growth suppression with significant activity at a concentration of 1 nM. The IC_50_ concentrations were 0.34 nM and 5.2 nM in T47D and MCF7 cells, respectively, which were lower compared with fulvestrant (fulvestrant IC_50_ was 2.2 nM and 19 nM in T47D and MCF7 cells, respectively) (Figure 1, A and B, and Supplemental Figure 1, A–C; supplemental material available online with this article; https://doi.org/10.1172/jci.insight.188051DS1). Off-target effects were assessed in the triple-negative breast cancer cell line, MDAMB231, in which imlunestrant did not affect cell proliferation (Supplemental Figure 1D). Given the importance of the pure antagonistic activity of a SERD, we tested the effects of imlunestrant in MCF7 cells overexpressing ERE-luciferase in apo and ligand-bound states (Figure 1, C–E). We observed strong antagonistic activity of imlunestrant in E2-treated conditions already after 6 hours of treatment, without evidence of agonistic activity in both states when testing imlunestrant at a concentration up to 1,000 nM and up to treatment for 24 hours. Similar results were seen with fulvestrant (Supplemental Figure 1F). In aggregate, these results indicate that imlunestrant is an ER antagonist without agonistic activity in breast cancer cells.

Next, we examined the ability of imlunestrant to degrade WT and Y537S ER by immunoblotting. Initial degradation of WT-ER was observed as early as 6 hours with the magnitude of ER degradation continuously increasing up to 48 hours and retained at 72 hours. Although, at 24 hours, the degradation of WT-ER was overall comparable between fulvestrant treatment and imlunestrant, mutant-ER protein degradation was superior with imlunestrant treatment versus fulvestrant (Figure 1, F and G). Furthermore, mutant ER degradation persisted beyond 24 hours with imlunestrant treatment. Despite the evidence of mutant and endogenous WT-ER degradation by imlunestrant and fulvestrant within 24 hours, the dose-response curves of imlunestrant and fulvestrant on day 5 shifted with the expression of the Y537S ER mutations (Figure 1B and Supplemental Figure 1, A–C). Longer term in vitro experiments with colony assays of WT and mutant ER cells showed that, at day 14, higher concentrations of imlunestrant were able to suppress cell confluency in the presence of the ER mutation (Figure 1, H and I). Taken together, these in vitro studies suggest that imlunestrant effectively degrades mutant ER; however, the ER mutations likely diminish the antagonistic activity of imlunestrant and this can be circumvented by a higher concentration.

**Figure 2:**
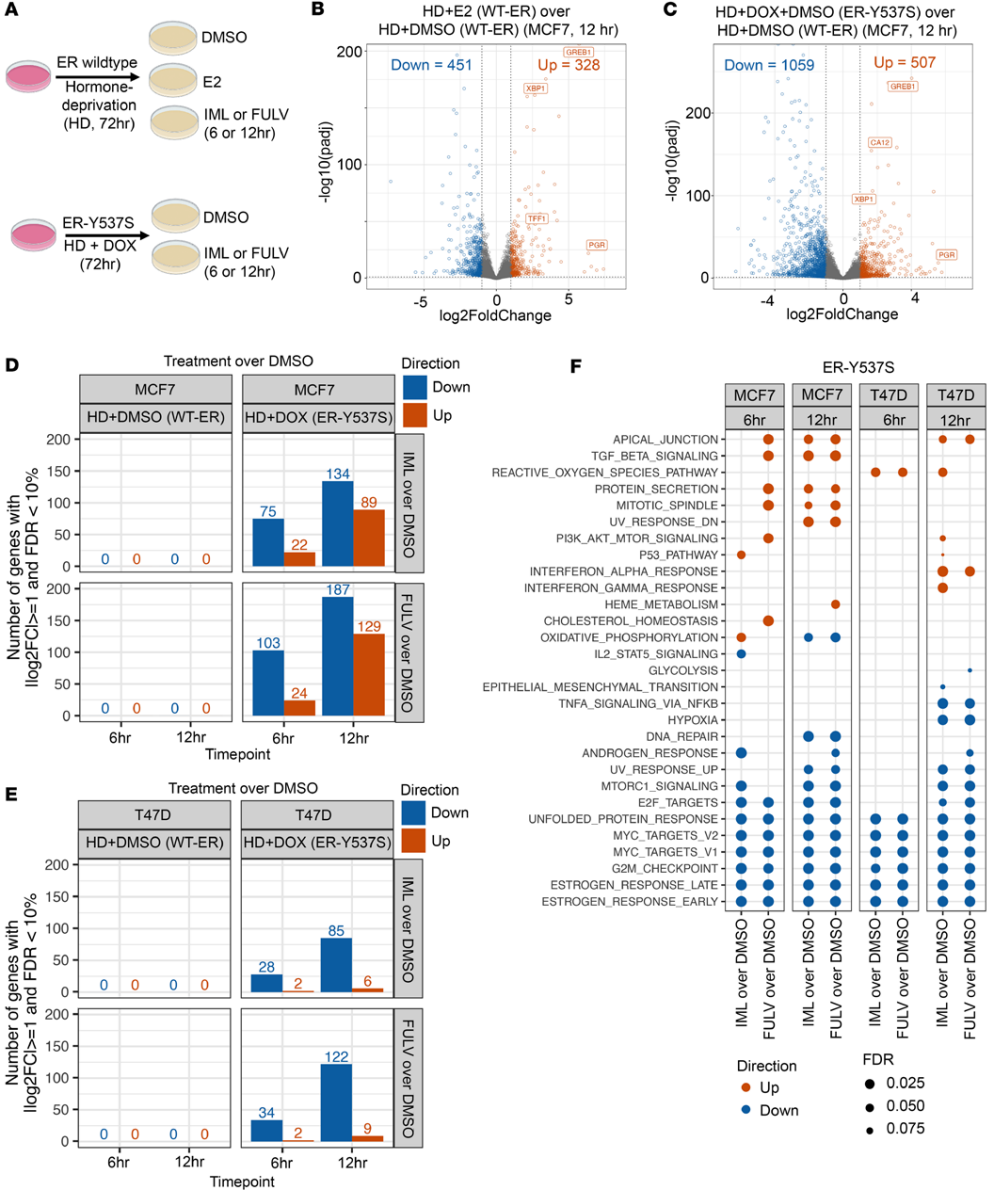
Transcriptional effects of imlunestrant. (**A**) Cartoon schematic of the RNA-seq treatment conditions. (**B**) Volcano plot of the differentially expressed genes following estradiol treatment or (**C**) doxycycline induction of ER-Y537S in MCF7 cells. Dotted lines denote chosen cutoffs |log_2_FC| ≥ 1 and FDR < 10%. Orange is upregulated and blue is downregulated following treatment. *n* = 2 replicates per group. ESR1 and estrogen-regulated genes are labeled with gene names when significant. (**D**) Bar plot of the number of differentially expressed genes in MCF7 or (**E**) T47D cells following IML or FULV treatment in cells expressing WT-ER or ER-Y537S. (**F**) Dot plot of gene set testing results in ER-Y537S-expressing cells for the Hallmark gene collection using cameraPR (10% FDR threshold per column).

To test for the selective activity of imlunestrant on transcription in a nonbiased, genome-wide manner, we performed RNA-seq experiments in hormone-deprived conditions without and with the induction of the Y537S ER mutation. The DOX-inducible cells were cultured in hormone-deprived (HD) conditions for 3 days and then treated with either vehicle (DMSO), E2, imlunestrant, or fulvestrant for 6 and 12 hours. Additionally, cells were first stimulated with DOX to induce the Y537S ER mutation followed by culture in HD conditions and then treatment with DMSO, imlunestrant, or fulvestrant for 6 and 12 hours (Figure 2A and Supplemental Table 1). As expected, E2 treatment of HD cells led to significant transcriptional changes, including the upregulation of canonical ER transcriptional targets (Figure 2B). In addition, induction of the Y537S ER mutation in HD conditions resulted in significant gene expression changes, including canonical ER transcription targets among other genes (Figure 2C), as we previously described (13). We next tested the transcriptional effects of imlunestrant and fulvestrant. In cells with WT ER after culture in HD conditions, imlunestrant and fulvestrant had no transcriptional effects, since ER is inactive in these conditions, highlighting the specificity of these two ER antagonists. In contrast, in MCF7 and T47D cells expressing the Y537S ER mutation in HD conditions, imlunestrant and fulvestrant treatment resulted in significant transcriptional changes, including the down regulation of genes of the estrogen response and cell cycle pathways (Figure 2, D and E, Supplemental Figure 2, A–D, and Supplemental Table 2). Overall, when comparing the pathways modulated by imlunestrant and fulvestrant treatment at 6 and 12 hours in MCF and T47D cells expressing the Y537S ER mutation in HD conditions, we observed comparable effects. Both drugs led to the downregulation of expected pathways, such as estrogen response early and late, E2F targets, G2M checkpoint, and MYC targets (Figure 2F). In aggregate, our results revealed highly selective on-target activity of imlunestrant in the presence of WT and mutant ER.

### Increased cell cycle arrest results in superior suppression of tumor growth.

**Figure 3:**
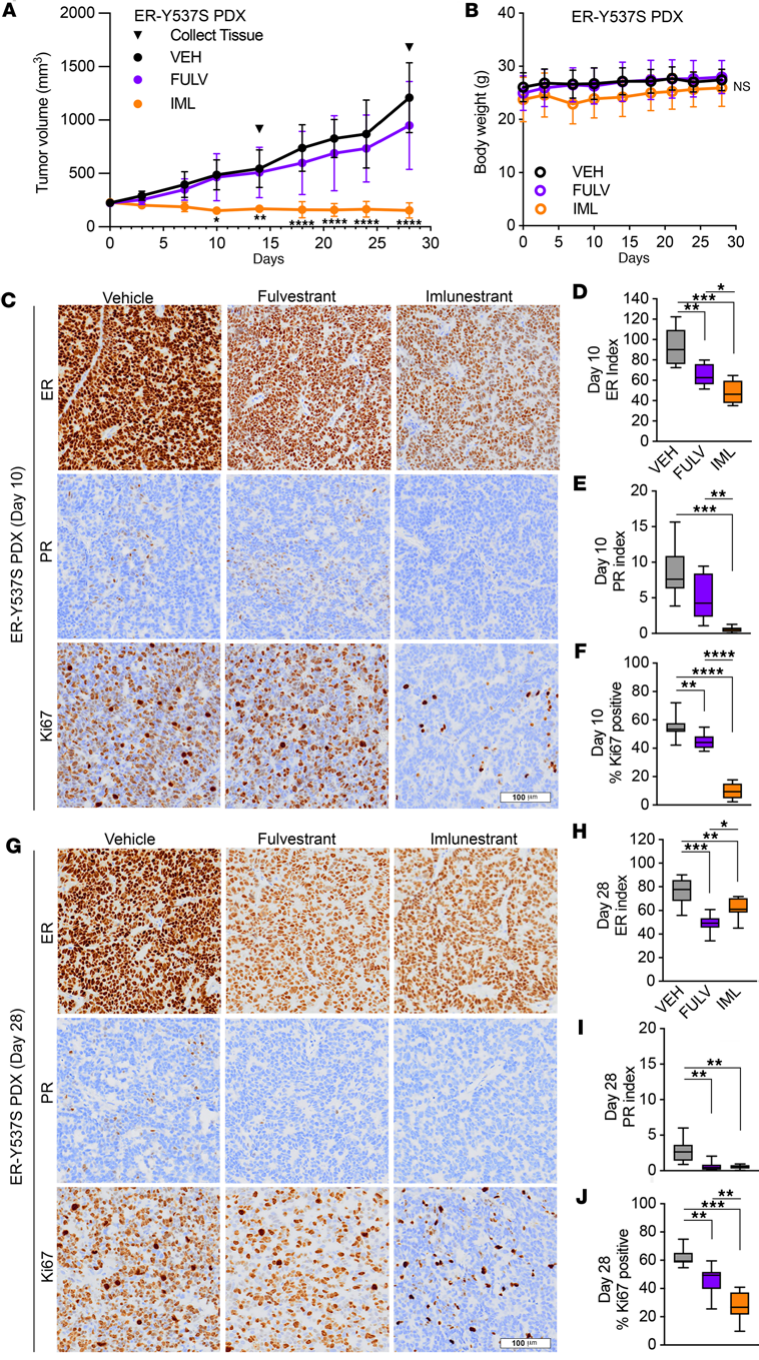
Imlunestrant treatment leads to decreased proliferation and tumor regression. (**A**) Tumor volumes of ER-Y537S PDX treated with vehicle (VEH, black), 5 mg subcutaneous fulvestrant (FULV, purple) per week, or 15mg/kg oral daily imlunestrant (IML, orange). Error bars denote ± SD. Two-way ANOVA with Dunnett’s multiple comparisons test. *n* = 3 mice/treatment on day 10 and *n* = 5 mice/treatment on day 28. (**B**) Body weight of the PDX-bearing mice treated with VEH, FULV, or IML. Error bars denote ± SD. One-way ANOVA with Tukey’s multiple comparisons test. (**C**) Representative images of IHC staining on ER-Y537S PDX day 10 for ER or PR or Ki67; scale bar: 100 μm. (**D**) IHC staining index for ER or (**E**) PR or (**F**) % Ki67 positive on ER-Y537S PDX day 10 tissue. Box and whisker plot with maximum and minimum. One-way ANOVA with Tukey’s multiple comparisons test, *n* = 2 mice, 4–5 images from each mouse. (**G**) Representative images of IHC staining on ER-Y537S PDX tissue treatment day 28 stained for ER or PR or Ki67. 20× magnification, scale bar: 100 μm. (**H**) IHC staining index for ER or (**I**) PR or (**J**) % Ki67 positive on day 28 ER-Y537S PDX tissue. Box and whisker plot with maximum and minimum. One-way ANOVA with Tukey’s multiple comparisons test, *n* = 2 mice, 4–5 images from each mouse. *P < 0.05, **P < 0.01, ***P < 0.001, ****P < 0.0001.

To evaluate the in vivo activity of imlunestrant, we turned to a PDX model derived from a liver metastasis from a patient who was heavily pretreated. This PDX has high ER expression, harbors a Y537S *ESR1* mutation, and is E2 independent (18). As shown in Figure 3A, the tumors were resistant to fulvestrant but were highly sensitive to imlunestrant. Already at day 10, treatment with imlunestrant as a single agent led to significant tumor regression, which persisted until the end of the experiment, at day 28. The mice tolerated imlunestrant treatment well without a significant weight change (Figure 3B). In line with the tumor growth results, ER, PR, and Ki67 levels were significantly lower in tumors treated with imlunestrant compared with fulvestrant and vehicle control at day 10 (Figure 3, C–F). Although, at day 28, in the residual tumors, ER levels were marginally higher in imlunestrant compared with fulvestrant treatment, Ki67 levels remained significantly lower with imlunestrant treatment (Figure 3, G–J).

**Figure 4:**
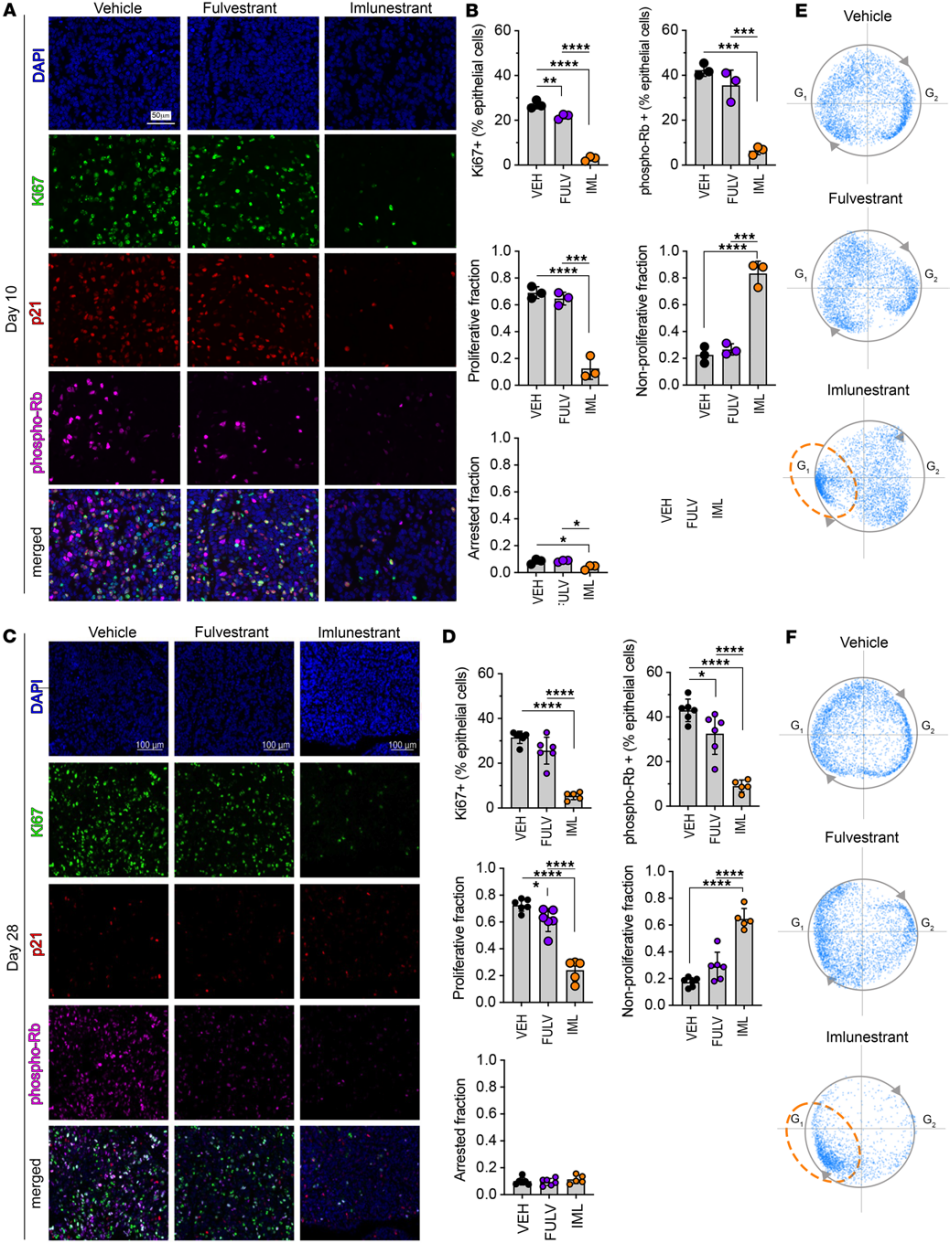
Multiparametric immunofluorescence analysis. (**A**) Representative immunofluorescence images for DAPI, Ki67, p21, Rb phosphorylated at Serines 807/811 (phospho-Rb), and merged staining from ER-Y537S patient-derived xenograft (PDX) treated for 10 days with vehicle, fulvestrant, or imlunestrant. *n* = 3 mice/treatment. Scale bar: 50 μm. (**B**) Multivariate proliferation index (MPI) quantification of proliferative fraction, nonproliferative fraction, and arrested fraction of epithelial cells after treatment with VEH (vehicle), FULV (fulvestrant), or IML (imlunestrant) for 10 days. Each point represents 1 mouse. Bar plot with average ± SD. One-way ANOVA with Tukey’s multiple comparisons test. (**C**) Representative immunofluorescent images after 28 days of treatment. *n* ≥ 4 mice/treatment. Scale bar: 100 μm. (**D**) MPI quantification of immunofluorescence after treatment for 28 days. One-way ANOVA with Tukey’s multiple comparisons test. (**E**) Pseudotime circle-fitted distribution of proliferative cells from ER-Y537S PDX tumors treated with VEH, FULV, or IML for 10 days or (**F**) 28 days. Scatter plot, each point represents a cell with *n* ≥ 1,909 cells/treatment. Orange circle indicates G1 cell cycle arrest in IML treated tumors. *P < 0.05, **P < 0.01, ***P < 0.001, ****P < 0.0001.

Next, we performed multiplexed immunofluorescence to calculate the Multivariate Proliferation Index (MPI) on PDX tumors harvested after treatment for 10 days (an early time point to assess direct effects) and 28 days. MPI is calculated based on expression of several distinct proliferative markers, including Ki67, PCNA, MCM2, and the 2 markers of cell-cycle arrest p21 and p27. MPI classifies each cell as either proliferative, if the cell expresses a positive balance of the proliferation markers; nonproliferative; for a cell that lacks the expression of proliferative markers; and arrested, if the cell expresses 1 or 2 of the cell-cycle arrest markers, irrespective of the expression of the proliferation markers, providing a comprehensive measure of the tumor proliferation state at the single-cell level (19). Imlunestrant treatment resulted in a significantly greater suppression of Ki67 and phospho-Rb compared with fulvestrant. Remarkably, on days 10 and 28, the percentage of proliferating cells was significantly decreased by imlunestrant, the nonproliferating tumor cells (Ki67- and phospho-Rb-) were significantly increased, and there was no impact of treatment on percentage of arrested cells (Figure 4, A–D). To further investigate the impact of imlunestrant on the cell cycle, the distribution of the cells was separated by pseudotime. In vehicle-treated tumors, the cells were equally distributed across the cell cycle in pseudotime. While fulvestrant treatment partially suppressed cell cycle progression, imlunestrant nearly completely blocked the cell cycle and cells accumulated in one area of pseudotime, which is most consistent with the G1 cell cycle phase (Figure 4, E and F).

**Figure 5:**
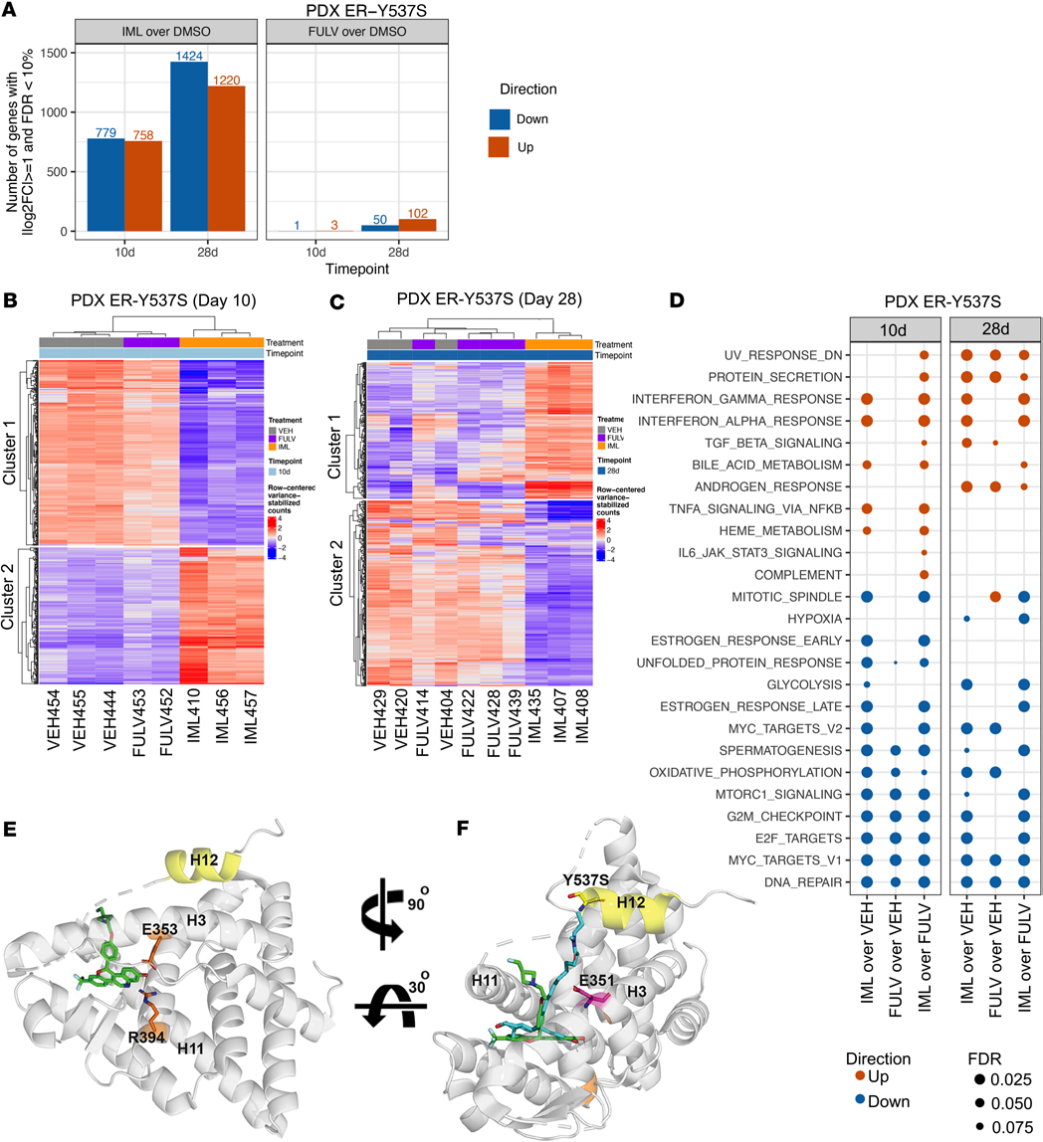
Transcriptomic effects of imlunestrant. (**A**) Bar plot of the number of differentially expressed genes in ER-Y537S patient-derived xenograft (PDX) treated with fulvestrant (FULV) or imlunestrant (IML) versus vehicle (VEH). Bars are orange for upregulated genes and blue for downregulated genes. Treatment duration for 10 days or 28 days. *n* ≥ 3 mice per group. (**B**) Heatmap of the row-centered variance-stabilized counts for the top 500 variable genes in ER-Y537S PDX tumors treated for 10 days with VEH, FULV, or IML. k-medoids clustering on 2 clusters with predominantly IML-downregulated genes and IML-upregulated genes following treatment for 10 days or (**C**) PDX treatment for 28 days. (**D**) Dot plot of gene set testing results for the Hallmark gene collection using cameraPR (10% FDR threshold per column). (**E**) Imlunestrant (green) docked to x-ray crystal structure of the ER LBD harboring the Y537S mutation (PDB: 9bu1). Proximity to residues important for ligation (orange) reveals imlunestrant occupation of ligand binding pocket. (**F**) Ligand binding pose comparison between imlunestrant and a derivative of the aliphatic SERD ICI 164,384 (PDB: 7r62, a derivative of estradiol that is closely related to fulvestrant) in cyan illustrates side arm conformation in relation to D351 (magenta) and S537 mutation (yellow).

The PDX tumors treated for 10 and 28 days with vehicle control, fulvestrant, and imlunestrant were subjected to RNA-seq analysis. Differential gene expression comparing treatment with vehicle control and imlunestrant or fulvestrant revealed a higher number of significant changes with imlunestrant versus fulvestrant treatment on day 10 and day 28 (Figure 5A and Supplemental Table 3). In addition, K-medoids (KM) clustering of the RNA-seq as early as day 10 and after 28 days detected 2 main gene clusters that segregated the tumors treated with imlunestrant from fulvestrant and vehicle control, highlighting the differences between these two ER antagonists in vivo (Figure 5, B and C). Pathway analysis showed that, overall, imlunestrant and fulvestrant impacted similar pathways, such as the downregulation of pathways related to estrogen response and the cell cycle. Additionally, there were a number of pathways that were uniquely modulated by imlunestrant, as an example, the upregulation of NFKB signaling and IFN-γ response, a pathway that was reported to be regulated by ET (20), was observed only with imlunestrant in this model. However, direct comparison of the tumors after imlunestrant and fulvestrant treatments at both time points showed that imlunestrant had more robust effects on the Hallmark pathways of cell cycle, estrogen response late (see Methods) and MYC targets pathways (Figure 5D). Taken together, these results demonstrate that, in vivo, better ER targeting with imlunestrant results in superior cell cycle suppression and enhanced tumor regression.

### Molecular docking in the presence of the Y537S mutant ligand binding domain reveals differences between imlunestrant and fulvestrant.

SERDs classically function by competitively binding to the ER ligand binding domain orthosteric hormone binding pocket. This action favors helix 12 binding within the activating function 2 (AF-2) cleft, blocking coactivator binding and increasing the dynamics of this region to promote proteasomal degradation of the receptor (21). Since imlunestrant exhibited superior Y537S *ESR1* mutant protein degradation and enhanced suppression of tumor growth in the Y537S *ESR1* mutant PDX model compared with fulvestrant, even with a fulvestrant dose considered above the clinically relevant therapeutic range (22), we hypothesized that imlunestrant impacts helix 12 differently than fulvestrant.

Due to our inability to obtain diffraction-quality crystals to understand the structural basis of imlunestrant’s and fulvestrant’s activities, we employed molecular docking to the Y537S ER ligand binding domain to test this hypothesis. At the most favorable binding energies, imlunestrant was predicted to occupy the orthosteric hormone binding pocket of ER LBD Y537S. Interestingly, a bifurcated E353 and R394 hydrogen bond is predicted like other SERDs, however, it was not predicted to bind D351 (Figure 5E).

We then sought to compare this predicted structure to that of fulvestrant. However, no cocrystal structure has been published to date. To address this, we aligned imlunestrant with a desmethyl derivative of ICI 164,384, which is an estradiol derivative chemically similar to fulvestrant (PDB: 7R62) (23). In this case, the aliphatic ICI molecule appears to directly perturb helix 12, while imlunestrant is predicted to mobilize the preceding loop. This destabilization of the loop that connects helix 11 to helix 12 has been previously shown to be sufficient to induce proteasomal degradation of the receptor (21, 24) (Figure 5F). Taken together, these data suggest that imlunestrant is likely to target the Y537S mutant ER ligand binding domain structural features that are different from fulvestrant.

### Genome-wide CRISPR screen to detect potential novel therapeutics in combination with imlunestrant.

**Figure 6:**
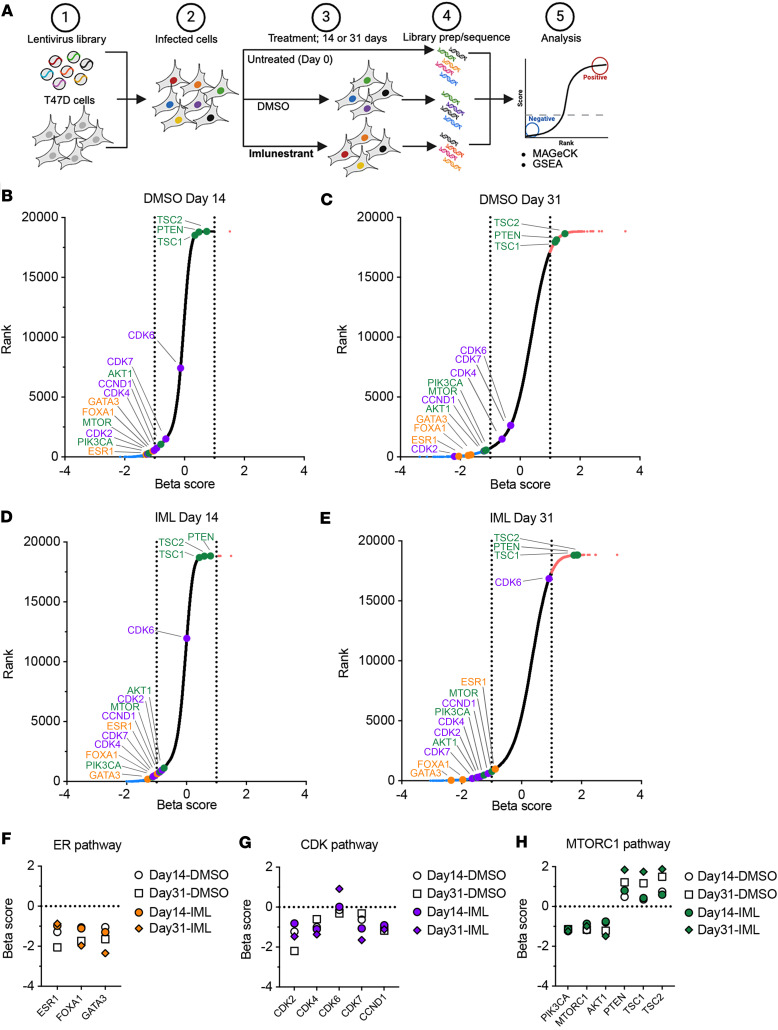
Genome-wide CRISPR screen in the presence of imlunestrant treatment. (**A**) Cartoon schematic of the genome-wide CRISPR/Cas9-knockout (KO) screen; (step 1) T47D cells are infected with the H3 lentiviral library of gRNAs, (step 2) infected cells are selected using puromycin, (step 3) cells are collected for untreated baseline (Day 0), or treated with DMSO or 1 nM imlunestrant, (step 4) after treatment for 14 or 31 days cells are collected, gRNAs amplified, and sequenced, followed by (step 5) gRNA analysis using MAGeCK algorithm. (**B**) MAGeCK β score and ranking results for gRNAs enriched or depleted after treatment with DMSO versus Day 0 for 14 days or (**C**) 31 days. (**D**) MAGeCK β score and ranking results for gRNAs enriched or depleted after treatment with imlunestrant versus Day 0 for 14 days or (**E**) 31 days. Scatter plot with each dot represents a gene in the genome-wide gRNA library, red are enriched after treatment (β ≥ +1), blue are depleted after treatment (β ≤ –1), black are insignificant after treatment, genes in the ER pathway are orange, the CDK pathway are purple, and the MTORC1 pathway are green. (**F**) β scores for individual genes depleted after imlunestrant treatment in the ER pathway, (**G**) the CDK pathway, or (**H**) the MTORC1 pathway.

We performed a genome-wide CRISPR/Cas9-knockout screen with the following aims: to further test the on-target effects of imlunestrant and to identify acquired vulnerabilities in the presence of imlunestrant that are putative therapeutic targets in combination with imlunestrant, which could potentiate the activity of a single-agent SERD. Cells induced with the genome-wide CRISPR/Cas9 library were treated with DMSO or imlunestrant and collected on days 0, 14, and 31 (at which point there were 10 cell doublings), followed by the analysis of gRNA-positive or -negative selection using the Mageck algorithm (25) (Figure 6A). Essentiality was determined based on the day 31 results and we included the results of day 14 to illustrate the kinetics of the screen (Supplemental Table 4). As expected, we detected positive enrichment of gRNAs targeting tumor suppressors, PTEN, TSC1, and TSC2, in both DMSO- and imlunestrant-treated cells on days 14 and 31, indicating that loss of PTEN and TSC1/2 enhance tumor cell growth without and with imlunestrant treatment (Figure 6, B–E). Conversely, we found that the essentiality score (β-score) of several genes with key functions in HR+ breast cancer was different after imlunestrant treatment and DMSO control conditions (both compared with day 0). The *ESR1* gene was not significantly essential after 31 days of imlunestrant treatment (β-score, –0.88), whereas in cells treated with DMSO for 31 days ESR1 was essential (β-score, –2.05) (Figure 6E). This result suggests that, with long-term treatment with imlunestrant, ER is not essential in providing a signal of adaptation to the treatment and supporting the on-target activity of imlunestrant. Interestingly, after 31 days of treatment with imlunestrant or DMSO, the essentiality score of *FOXA1* increased, whereas the essentiality score of *GATA3* increased only after imlunestrant treatment (Figure 6F). These different dynamics in the essentiality scores highlight the unique functions of these lineage-defining transcription factors. Genes related to the cyclin D1-Rb1 axis, such as *CCND1*, *CDK4*, and *CDK2* were among the most essential genes in DMSO-treated cells. These genes remained essential after imlunestrant treatment, albeit with lower essentiality scores when compared with DMSO treatment. Notably, CDK7, a master regulator of the cell cycle and transcription, and an essential gene in ER-Y537S cells and CDK4/6i resistance (13, 18), was essential with imlunestrant treatment (Figure 6G). In addition, a number of genes that were essential in DMSO-treated cells that can be targeted by drugs that are approved for the treatment of HR^+^ breast cancer retained essentiality after imlunestrant treatment, such as *PIK3CA*, *MTOR1*, and *AKT1* (26–28) (Figure 6H). In summary, these results validate our CRISPR screening approach, highlight the on-target activity of imlunestrant, and offer potential therapeutic combinations to enhance the activity of imlunestrant.

**Figure 7:**
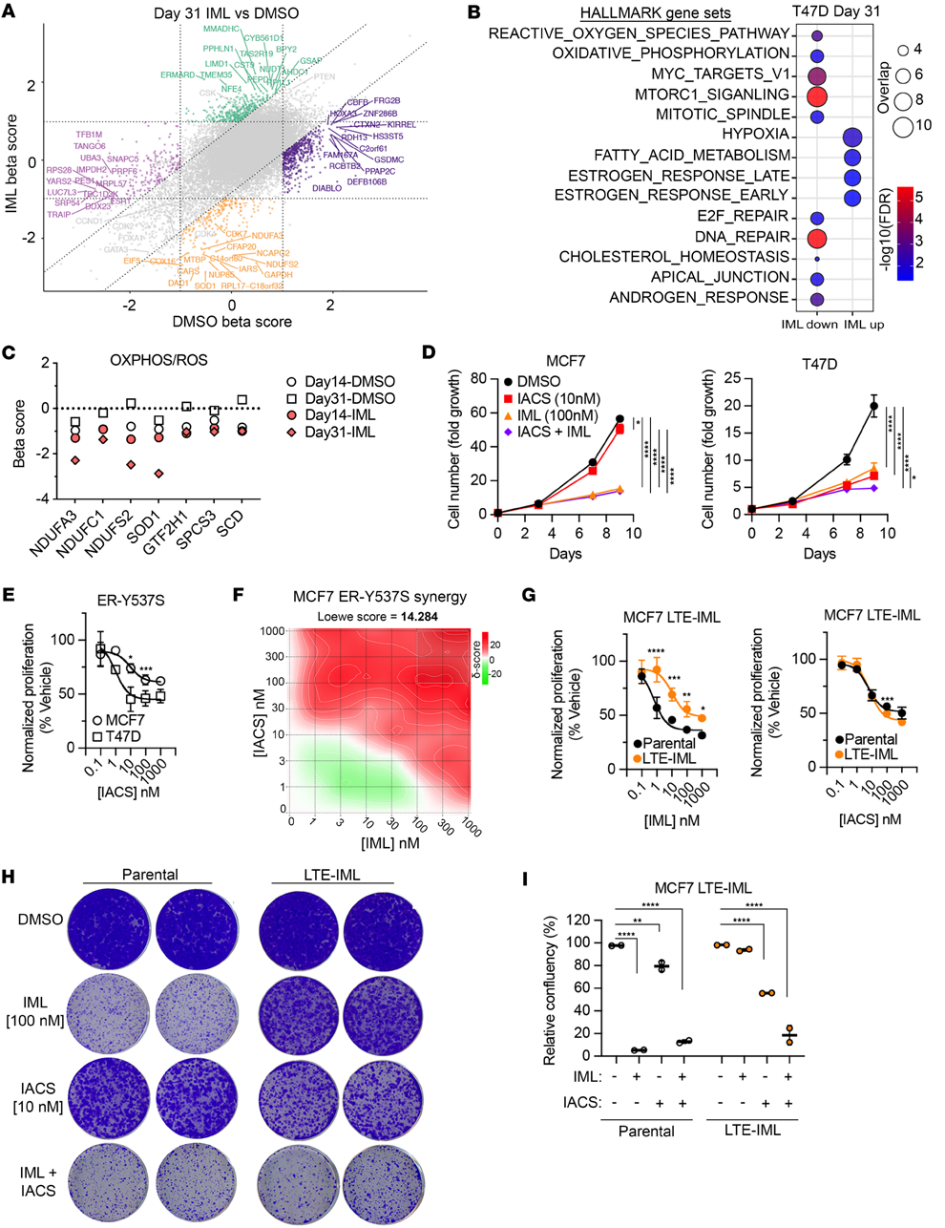
Imlunestrant treatment increases the essentiality of genes related to oxidative phosphorylation. (**A**) Nine-square scatter plot of the gene β scores in day 31 IML or day 31 DMSO treated T47D cells compared with day 0. Vertical and horizontal dotted lines denote ± 1 SD of DMSO or IML β scores, respectively, and diagonal dotted lines denote ± 1 SD of the difference in β scores (IML – DMSO). Green are gRNAs enriched in only IML (IML up; IML β score > 1 and DMSO β score < 1) and orange are gRNAs depleted only in IML (IML down; IML β score < –1 and DMSO β score > –1 and < 1). (**B**) Balloon plot of Hallmark GSEA results for genes in IML up or IML down. Circle size is the number of overlapping genes and circle color is the FDR q-value. (**C**) Individual gene β scores that are depleted in IML treated cells from the HALLMARK_OXIDATIVE_PHOSPHORYLATION and HALLMARK_REACTIVE_OXYGEN_SPECIES pathways. (**D**) Fold change for MCF7 (left) or T47D (right) cells during treatment up to 8 days with DMSO, IACS (10 nM), IML (100 nM), or IACS and IML. Growth curve with average ± SD. One-way ANOVA with Tukey’s multiple comparisons test. (**E**) Normalized cell proliferation for MCF7 (circle) or T47D (square) cells expressing ER-Y537S and treated with a dose-response of IACS for 5 days. Growth curves with average ± SD. Two-way ANOVA with Šidák’s multiple comparisons test. (**F**) Synergy distribution analysis in MCF7 ER-Y537S cells treated with a dose response matrix of IACS and IML. Loewe synergy score using the synergyfinder tool. Red indicates synergism. (**G**) Normalized cell proliferation for MCF7 parental (black) or MCF7 LTE-IML (orange) cells treated with IML (left) or IACS (right). Growth curve with average ± SD. Two-way ANOVA with Šidák’s multiple comparisons test. (**H**) Colony assay crystal violet staining results from MCF7 parental or LTE-IML cells treated with imlunestrant (100 nM), IACS (10 nM), IML and IACS for 2 weeks in full media. (**I**) Relative confluency of colony assay crystal violet staining in **H**. Bar graph with average ± SD. Two-way ANOVA with Dunnett’s multiple comparisons test. *P < 0.05, **P < 0.01, ***P < 0.001, ****P < 0.0001.

We next compared the essentiality scores (β-score) of the CRISPR screens after 31 days of DMSO control and imlunestrant (Figure 7A). This comparison identified a set of genes that gained essentiality only after imlunestrant treatment (lower β-scores) or decreased dependency (higher β-scores). Pathway analysis revealed that the genes with increased essentiality are involved in pathways that are known to be associated with resistance to endocrine therapy, such as MTOR1 signaling and MYC targets (3, 29) (Figure 7B). Interestingly, multiple genes related to reactive oxygen species (ROS) and oxidative phosphorylation pathway (OXPHOS) were among the top-ranked genes that gained essentiality after 31 days of imlunestrant treatment (Figure 7, B and C). The OXPHOS metabolic pathway enables the production of ATP by the transport of electrons through a series of transmembrane protein complexes in the mitochondrial membrane. ROS are byproducts of the OXPHOS pathway, and, therefore, these pathways are highly connected. Several cancers were shown to be reliant on OXPHOS, and upregulation of OXPHOS was shown to be a mechanism of drug resistance in several cancers (30, 31). Specifically, the genes with the most significant β-score in these pathways that also gained essentiality after imlunestrant treatment included SOD1, NDUFSA3, NDUFC1,and NDUFS2 (Figure 7C). These genes are critical components of mitochondrial energy metabolism and cellular redox balance. SOD1 is a Cu/Zn superoxide dismutase that plays a major role in redox balance and protects against superoxide radicals (32). NDUFA3 (NADH:ubiquinone oxidoreductase subunit A3 and C1) is predicted to be involved in assembly of complex I in the mitochondrial OXPHOS respiratory chain, and NDUFC1 and NDUFS2 are complex I core components. Taken together, these genes are key components of redox balance and mitochondria OXPHOS, and these results provide a rationale for testing inhibitors of these pathways, particularly inhibitors of the OXPHOS complex 1, in combination with imlunestrant.

To follow up on our CRISPR screen results and test the effect of pharmacological inhibition of OXPHOS, we used the small molecule inhibitor of OXPHOS complex I, IACS-010759 (IACS) (33). We did not observe a substantial effect on growth after 9 days of treatment with IACS alone at a concentration of 10 nM or when IACS was added to imlunestrant in MCF7 cells (Figure 7D). In contrast, in T47D cells, IACS alone suppressed cell growth and there was a small added effect when combined with imlunestrant. In the MCF7 and T47D endocrine-resistant cells expressing the Y537S *ESR1* mutation, we observed a dose-response effect with IACS in both MCF7 and T47D cells, and again with superior activity in the T47D cells (Figure 7E). Importantly, there was significant synergy between IACS and imlunestrant in the MCF7 cells expressing the Y537S mutation (Figure 7F). Finally, imlunestrant-resistant cells were generated by culturing MCF7 in the presence of escalating doses of imlunestrant. As expected, imlunestrant-resistant cells displayed an increased IC_50_ and increased confluency of colonies after imlunestrant treatment (Figure 7, G–I). While IACS alone already resulted in a significant decrease in the colony confluency in the imlunestrant-resistant cells, the addition of imlunestrant to IACS enhanced this effect (Figure 7, H and I). In aggregate, these results provide evidence for the tumor-suppressive effect of OXPHOS complex I inhibition in combination with imlunestrant as a potential strategy to enhance the activity of imlunestrant and overcome resistance to imlunestrant in ER^+^ breast cancer.

## Discussion

SERDs have several potential advantages compared with SERMS and aromatase inhibitors. Specifically, the pure antagonistic activity of SERDs that precedes the increase in the turnover of ER is a key feature that differentiates this class of drugs from SERMS, which have context-dependent agonistic activity in addition to their antagonistic activity (34). In addition, the ability of SERDS to enhance ER degradation can potentially circumvent ligand-independent activity, a key mechanism of endocrine resistance in which ER remains a key tumor driver.

Fulvestrant, the first-in-class SERD, is limited by its poor bioavailability, requiring intramuscular injections and poor pharmacokinetics. The CONFIRM clinical trial showed that a higher dose of fulvestrant (500 mg versus 250 mg) resulted in superior clinical outcomes (35, 36), but FES-PET imaging indicated incomplete ER antagonism with fulvestrant treatment even at the higher doses (37). More recently, retrospective analyses of clinical trials indicate that the presence of the LBD activating ER mutations confer resistance to fulvestrant (12). Taken together, these limitations of fulvestrant provide a rationale for developing next-generation oral SERDs with improved pharmacokinetics and activity against the *ESR1* mutations.

Imlunestrant is an oral SERD in clinical development. Preclinical studies also demonstrated that imlunestrant is brain penetrant (38). The phase I/1b EMBER study (NCT04188548) investigating imlunestrant as a single agent and in combination with other targeted agents established the recommended phase 2 dose (RP2D) of imlunestrant (17). The phase III EMBER 3 clinical trial investigated imlunestrant versus fulvestrant versus imlunestrant plus abemacilib in patients with advanced ER^+^ breast cancer who have progressed on first-line endocrine therapy–based treatment and showed that imlunestrant had superior activity compared with fulvestrant in patients with an *ESR1* mutation (39). The phase III EMBER 4 clinical trial (NCT05514054) is investigating adjuvant imlunestrant versus standard-of-care endocrine therapy after 2 years and within 5 years of adjuvant treatment. In addition to imlunestrant, there are several other next generation oral endocrine therapies that are either already approved (elacestrant) or are in advanced phases of clinical development (camizestrant, vepdegestran,t and giredestrant) (16). We did not investigate how these other agents compare with imlunestrant, which is a limitation of our study.

In this study, we investigated the preclinical pharmacology and efficacy of imlunestrant in WT and hormone-resistant mutant ER and demonstrated several clinically relevant characteristics of imlunestrant. Our studies focused on the Y537S ER mutation, since it has been shown to have the most robust phenotypes, including both its constitutive and antagonistic activity (13). Imlunestrant exhibited pure antagonistic activity preceding the degradation activity. In addition, imlunestrant leads to ER degradation in the presence of WT and mutant ER. Compared with fulvestrant, imlunestrant demonstrated superior degradation of the Y537S mutant ER, which could potentially be explained by the differences in the molecular docking of these two agents in the presence of the mutant ligand binding domain. Remarkably, we demonstrated the highly specific on-target activity of imlunestrant, as evidenced by the absence of gene expression changes when treating cells in which ER transcriptional activity was suppressed by hormone deprivation.

In the in vitro studies, we did not observe superior tumor cell growth suppression with imlunestrant compared with fulvestrant in the presence of the Y537S ER mutation. However, our in vivo study in a PDX model harboring a Y537S mutation demonstrated that improved ER blockade with imlunstrant led to enhanced tumor suppression with evidence of tumor regression, despite resistance to a relatively high dose of fulvestrant that is considered above the clinically relevant therapeutic dose (22). Molecular analysis of the tumors at early points showed enhanced ER degradation with imlunestrant versus fulvestrant. Importantly, comprehensive analysis of the cell cycle using Ki67, the MPI algorithm, and transcriptomic data, provides evidence that, in the presence of an *ESR1* mutation, better ER targeting is associated with increased cell cycle inhibition and potentiates tumor growth suppression. In addition, transcriptomic analysis showed that imlunestrant treatment resulted in increased expression of IFN-γ response and NFKB signaling, consistent with a previous study showing that ER blockade induces increased Rel A chromatin binding and NFKB signaling, which, in turn, increases the expression of genes involved in the IFN-γ pathway in a cell-intrinsic manner (20). This may have important therapeutic implications and warrants further preclinical investigation of the combination of SMAC mimetics (20), immune therapies, and imlunestrant in HR^+^ breast cancer models.

Our genome-wide CRISPR knockout screen during imlunestrant treatment showed that known vulnerabilities in HR^+^ breast cancer, such as genes related to the CDK4/6-RB1 axis and PI3Kinase signaling pathway, remain essential in the presence of imlunestrant treatment. This supports the clinical investigation of imlunestrant in combination with drugs targeting these pathways, such as CDK4/6i and PI3Kinase and AKT inhibitors. In addition, we identified a new vulnerability that is a potential therapeutic target. Several genes related to OXPHOS were among the top genes that gained essentiality during imlunestrant treatment. As a follow up of these results, we showed synergistic activity between imlunestrant and the OXPHOS inhibitor IACS in cells expressing the ER Y537S mutation. In addition, cells with acquired resistance to imlunestrant were sensitive to IACS. These results are in line with studies showing that, in more aggressive cancers, treatment resistance or metastases or there is a switch from aerobic glycolysis to OXPHOS for ATP generation (40). Moreover, recent studies also showed that, with the acquisition of endocrine resistance, there is a metabolic rewiring characterized by the dependency on OXPHOS (41, 42). Disappointingly, the phase I clinical trial of IACS failed to establish the recommended RP2D because of dose-limiting toxicities (43). However, this study investigated IACS as a single agent and it is possible that the addition of a low dose of IACS would be tolerated and could be utilized to enhance the activity of imlunestrant and prolong disease control. Nevertheless, these results unveil a key metabolic compensatory mechanism of resistance to imlunestrant and support further investigation of approaches to target this metabolic change for therapeutic purposes. We also identified CDK7 as one of the top-ranked vulnerabilities during imlunestrant treatment. In keeping with this finding, in previous studies, we identified CDK7 as a potential therapeutic target in ER^+^ breast cancer, and selective CDK7 inhibitors are currently in clinical development in metastatic HR^+^ breast cancer (44).

In summary, we provide preclinical evidence of imlunestrant’s selective on-target antagonistic activity. Importantly, imlunestrant as a single agent can overcome resistance engendered by the Y537S *ESR1* mutation. In addition, we identified several genes that gained essentiality during treatment with imlunestrant that can be targeted pharmacologically, offering a rationale for clinical testing of drug combinations, such as CDK4i, CDK7i, and PI3Kinase inhibitors with imlunestrant.

## Methods

### Sex as a biological variable.

Our study exclusively examined female mice because the disease modeled is mostly relevant in females, as male breast cancer is rare.

### Cell lines.

Cell lines were purchased from ATCC, independently validated using SPR testing, and routinely tested for mycoplasma contamination. Cells were maintained in sterile culture at 37°C and 5% CO_2_. MCF7 cells were grown in DMEM (Corning, 10013VC) supplemented with 10% FBS (Sigma, F2442), 1× Penicillin/Streptomycin (Corning, 3002CI), and 10 μg/mL recombinant human insulin (Sigma, I0516). T47D cells were grown in RPMI (Gibco, 11835055) supplemented 10% FBS, 1× Penicillin/Streptomycin, and 1% glutamine (Corning, 25005CI). HEK293T cells were grown in DMEM supplemented with 10% FBS, 1× Penicillin/Streptomycin, 1× sodium pyruvate (Gibco, 11360070), 1× nonessential amino acids (Gibco, 11140050), and 1× glutamine. As indicated, cells were hormone deprived (HD) for 72 hours by media change into phenol red–free media supplemented with 10% charcoal dextran-treated FBS. MCF7 cells with a doxycycline-inducible Y537S mutation (MCF7-Y537S) in ER and T47D cells with a doxycycline-inducible Y537S mutation (T47D-Y537S) were created as previously described (13). For doxycycline-inducible expression of ER-Y537S, cells were treated with 1 μg/mL doxycycline for 48–72 hours. To generate imlunestrant-resistant cells, MCF7 cells were cultured in increasing concentrations of imlunestrant (i.e., long term exposure [LTE]) starting from 1 nM and ending at 100 nM. Imlunestrant was provided by Lilly for most of the experiments except for the experiments in Figures 7, D–I. For these experiments imlunestrant was purchased from MedChem Express (HY-145572).

### ERE-LUC.

MCF7 cells stably expressing a luminescent reported under control of an estrogen response element (MCF7 ERE-LUC) were used to measure agonistic activity of ER ligands (45). Cells (1 × 10^3^) were plated in each well of a white opaque 96 well plate in at least quadruplicate. The next day, media was changed to HD media supplemented with the indicated treatment. After treatment, luminescence was measured using a microplate reader. For normalization, protein content was measured by addition of BCA reagent and colorimetric measurement using a microplate reader.

### Western blot.

Cells (2 × 10^6^) were plated in a 10 cm^2^ dish. The next day, the media was changed to HD media with or without doxycycline. Cells were treated as indicated for 24 hours followed by cell lysis in RIPA buffer supplemented with protease and phosphatase inhibitors. Protein concentrations were normalized using BCA assay, SDS-PAGE was performed using the BioRad mini electrophoresis system and 4%–12% NuPage Bis/Tris gels, proteins were transferred to nitrocellulose membranes using a BioRad mini Turbo, and blotted for estrogen receptor (CST, #D6R2W) or GAPDH (CST #14C10). Chemiluminescence was captured using a BioRad ChemiDoc, and densitometry quantification was performed on digital images in Fiji/ImageJ.

### Cell proliferation.

Cells (5 × 10^3^) were plated in each well of a 24 well plate in full media in quadruplicate. The next day, the media was changed to include the indicated treatment. After 5 days of treatment, cells were stained using X hoescht and X propidium iodide, followed by counting the live cell number (hoescht positive and propidium iodide negative) using Celigo automated imaging platform. Replicates were averaged and normalized to DMSO control.

### Colony growth.

Cells (5 × 10^3^) were plated in each well of a 6 well plate in full media in duplicate. The next day, the media was changed to include the indicated treatment. After 2 weeks of treatment, cells were fixed in ice-cold methanol, stained using 0.1% crystal violet in methanol, washed in water, and dried overnight. Images were captured using a digital scanner and the crystal violet area was quantified using Fiji/ImageJ.

### RNA-seq.

MCF7 or T47D cells (2 × 10^6^) were plated in 10 cm^2^ dishes in triplicate. The next day, media was changed to HD media with or without doxycycline. Cells were treated with estradiol (10 nM), imlunestrant (100 nM), fulvestrant (100 nM), or DMSO for 24 hours. Cells were washed, snap frozen in liquid nitrogen, and RNA was extracted using Trizol and RNeasy mini (Qiagen). RNA-seq was performed on the NovaSeqX platform. The VIPER pipeline was used for the alignment and quality control (46) of the samples. Alignment to the hg19 human genome was done with STAR v2.7.0 (47) and transcript assembly was done with cufflinks v2.2.1 (48). Quality control was done with RseQC v2.6.2 (49, 50). We assessed each sample on metrics of mappable reads, percentage of rRNA reads, gene body coverage, junction saturation and insert size for paired ends, and exploratory data analysis on variance-stabilized counts to determine samples that were of adequate quality. Differential expression testing was done using DESeq2 v1.44.0 (51) and ashr_v2.2-63 (52). Genes plotted in expression heatmaps were clustered by partitioning around medoids using the cluster v2.1.8 R package (https://CRAN.R-project.org/package=cluster). Genes were considered significantly differentially expressed if their ashr-shrunken log_2_ fold change was ≥ 1 with an adjusted *P* value < 0.10. Gene set testing on the Hallmark gene collection (53) was performed using the preranked version of correlation adjusted mean rank gene set test (cameraPR) using limma v3.60.6 (54, 55), where genes were preranked by their Wald statistics from DESeq2 testing. Multiple testing was accounted for using the Benjamini-Hochberg procedure by controlling at a 10% FDR. Analyses were performed using R v4.4.1 [R Core Team (2024). _R: A Language and Environment for Statistical Computing_. R Foundation for Statistical Computing, Vienna, Austria. <https://www.R-project.org>].

### Animal studies.

All mice were maintained in accordance with local guidelines and therapeutic interventions approved by the Animal Care and Use Committees of the Dana-Farber Cancer Institute. These patient-derived xenograft (PDX) details were published previously (18). Tumor fragments from the Y537S ER-mutant PDX1526 were dipped in 50% matrigel and implanted into the fourth mammary fat pads of ovariectomized NOD-SCID-IL2Rgc^–/–^ mice (Jackson Laboratories) without estradiol (E2) supplements. Anesthesia for the implantation was performed with isoflurane mixed with medical air. When tumors reached 150–200 mm^3^, mice were randomized into 3 groups (*n* = 5–8 mice per group): vehicle control (DMSO), fulvestrant 5 mg once a week subcutaneous, imlunestrant 15 mg/kg daily oral gavage for 28 days. Tumor volumes were measured at least once a week. An additional 3 mice per group were treated for 10 days only. After treatment, mice were euthanized and tumors were harvested. Mouse euthanasia was performed using carbon dioxide (CO_2_) followed by cervical dislocation. Tumors were halved for snap freezing for RNA extraction and the other half for fixing in formalin and paraffin embedding for IHC and immunofluorescence staining.

### IHC.

IHC was performed on the Leica Bond III automated staining platform using the Leica Biosystems Refine Detection Kit (Leica; DS9800). FFPE tissue sections were baked for 30 minutes at 60°C and deparaffinized (Leica AR9222) prior to staining. Primary antibodies were incubated for 30 minutes, visualized via DAB, and counterstained with hematoxylin (Leica DS9800). The slides were rehydrated in graded alcohol and coverslipped using the HistoreCore Spectra CV mounting medium (Leica 3801733). The slides were stained with the following antibodies: Ki67 from Biocare Medical, catalog number PRM325, clone SP6 was run at the ready to use concentration with a 20 M EDTA antigen retrieval (Leica ER2 AR9640), Progesterone Receptor from Dako, catalog number M3569, clone PgR 636 was run at 1:400 dilution with a 20 M EDTA antigen retrieval (Leica ER2 AR9640) and ER α from Neomarkers, catalog number RM-9101, clone SP1 was run at 1:40 dilution with a 30 M citrate antigen retrieval (Leica ER2 AR9961).

### CyCIF MPI.

The methods for CycIF and MPI calculation were previously described (19). Briefly, FFPE slides were baked at 60°C for 30 minutes, dewaxed using Bond Dewax solution at 72°C, and antigen retrieval was performed with Epitope Retrieval 1 solution at 100°C for 20 minutes using the BOND RX Automated IHC/ISH Stainer. Antibodies for each cycle were diluted in Odyssey Blocking Buffer and incubated overnight at 4°C in the dark. After antibody incubation, slides were stained with Hoechst 33342 for 10 minutes at room temperature. Slides were cover slipped using 20%–50% glycerol solution (Sigma-Aldrich, G5516) in PBS. Images were taken using DAPI, FITC, Cy3, and Cy5 channels either on the RareCyte CyteFinder (20x/0.75NA objective). After imaging, fluorophores were inactivated (4.5% H_2_O_2_, 20mM NaOH in PBS, 45 minutes) under LED lights, and the next cycle was performed. CyCIF image processing is organized in the following steps: stitching, registration, and correction of acquisition artifacts was performed using ASHLAR and the BaSiC algorithm. Ilastik software was trained on cropped images to label nuclear, cytoplasmic, and background areas. Pan-cytokeratin positivity was used as the threshold for determining epithelial cells, which are the cells included in the analysis. Data aggregation, filtering, normalization, and analysis were performed as previously described (19). Multivariate Proliferation Index (MPI) calculation; MPI is based on the normalized measurement of 5 markers: 3 proliferation markers (Ki-67 [CST #11882S], MCM2 [Abcam 223403], PCNA [CST# 8580S) and 2 cell cycle arrest markers (p21[CST 8493s], p27[ab206927]). The method avoids relying on single markers while separating cells expressing high level arrest markers (even if proliferation markers are expressed). The threshold values for proliferation and arrest are dataset dependent. Additional details and access to the underlying code can be found at https://github.com/labsyspharm/ashlar and https://github.com/santagatalab

### CRISPR screen.

For the CRISPR screen we used the H3 genome wide gRNA library (Addgene #133914) that targets 18,000 annotated genes in the human genome, with 6 gRNAs per gene on average for a total of 117,587 gRNAs and 3,842 control gRNAs targeting AAVS1, ROSA26, and CCR5. Lentiviral particles encoding the H3 genome wide gRNA library were produced by transient transfection of HEK239T cells using OptiMmem and Xtreme gene transfection reagent according to manufacture instructions. After 72 hours, the lentiviral supernatant was collected, cleared by centrifugation and syringe filter, followed by freezing aliquots at –80°C. For the CRISPR screen, T47D cells (2.7 × 10^8^) were transduced in suspension with 100 μg/mL polybrene at MOI = 0.3. Cells were selected in 1.5 μg/mL puromycin for 96 hours, cells were passaged, 3 × 10^6^ cells were collected for the day 0 sample, or 3 × 10^6^ cells were plated for DMSO or imlunestrant treatment. Cells were maintained in full growth media with 1 nM of imlunestrant or DMSO for 14 or 31 days. Cells were collected, washed, snap frozen in liquid nitrogen, and stored at –80°C. DNA was extracted using DNAeasy kit (Qiagen), gRNA barcodes were PCR amplified with adapters for sequencing on the Illumina platform. DNA libraries were sequenced at the DFCI Molecular biology core facility using the NextSeq5000 system. Sequencing results were analyzed using MAGeCK-VISPR (25) and MAGeCK-FLUTE v2.8.0 (R package version 2.8.0, <https://bioconductor.org/packages/MAGeCKFlute>).

### BLISS synergy assay.

MCF7 cells in full growth media were treated with doxycycline for 48 hours, then cells (1 × 10^3^) were seeded in each well of a 96 well plate at least in quadruplicate. The next day, cells were treated as indicated with imlunestrant, IACS, or DMSO. Cells were treated with an 8-point dose response matrix using 3-fold steps centered around each drug IC_50_. After treatment for 5 days, live cell number was counted using Celigo, and synergy quantified using BLISS synergy tool (https://synergyfinder.fimm.fi/synergy/20240901234814724196/).

### Molecular docking modeling.

Protein x-ray crystal structures were retrieved from RCSB PDB. Python Molecular Viewer was used to add polar hydrogens and Kollman Charges before docking the ligand over the ligand binding pocket using AutoDock Vina. Results were visualized using PyMOL. *Statistics*. Statistical analysis was performed in Prism 3.0 (Graphpad). Two-tailed Student’s *t* test was performed when comparing 2 groups and 2-tailed ANOVA was performed for multiple comparisons. *P* values less than 0.05 are considered significant and the corresponding statistical tests are reported in the figure legends.

### Study approval.

For PDX studies, patient consent for tumor implantation in nude mice was obtained under an IRB approved protocol (Dana-Farber/Harvard Cancer Center IRB protocol 93-085) and with patient consent and in compliance with the Declaration of Helsinki.

### Data availability.

RNA-seq data has been deposited to GEO database with the accession number GSE295024. All data values for all graphs are included in the Supporting Data Values file.

## Author contributions

S Sherman, ZMS, SWF, and RJ conceptualized the study and wrote the manuscript. S Sherman, DR, AV, DZ, GG, SK, and GK conducted the formal analysis. RJ acquired funding for the study. S Sherman, ZS, AN, CH, DF, GK, AV, and FHP completed the experiments and data collection. QDN, S Santagata, SWF, and RJ supervised the study. ZMS, DR, and AV prepared the figures for publication. All authors reviewed and edited the manuscript and approved the final manuscript.

## Supplementary Material

Supplemental data

Unedited blot and gel images

Supplemental tables 1-4

Supporting data values

## Version 1 05/06/2025

In-Press Preview

## Version 2 06/23/2025

Electronic publication
