# Supplementary material for: Imlunestrant a next-generation oral SERD overcomes ESR1 mutant resistance in estrogen receptor–positive breast cancer
Source: JCI Insight. 2025 May 6;10(12):e188051. doi: 10.1172/jci.insight.188051 (PMC12226049; doi:10.1172/jci.insight.188051)

SUPPLEMENTAL MATERIAL

Imlunestrant a next-generation oral SERD overcomes *ESR1* mutant resistance in estrogen receptor-positive breast cancer

Supplemental figures: 2

Supplemental Figure 1

A) Fold change for MCF7 (left) or T47D (right) cells expressing doxycycline inducible ER-Y537S and treatment up to 8 days with DMSO or imlunestrant (IML) at the indicated concentration. Growth curve with average ± standard deviation. Two-way ANOVA with Dunnett's multiple comparisons test. B) Normalized cell proliferation for MCF7 (left) or T47D (right) cells expressing ER wildtype or ER-Y537S and treated with a dose-response of fulvestrant for 5 days. Growth curve with average ± standard deviation. Two-way ANOVA with Sidak's multiple comparisons test. C) Cell proliferation assay IC_{50} values (nM) for imlunestrant or fulvestrant in MCF7 and T47D ER wildtype or ER-Y537S cells. D) Normalized luciferase signal in MCF7 ERE-LUC cells after hormone deprivation (HD) and treatment with or without E_2 (1 nM) and fulvestrant (0, 50 nM, 500 nM, or 1000 nM) for 1 hr (top), 6 hr (middle), or 24 hr (bottom). Bar graph with averages ± standard deviation. Two-way ANOVA with Tukey's multiple comparisons test. E) Fold change for MDAMB231 cells during treatment up to 6 days with DMSO or fulvestrant (FULV) at the indicated concentration. Scatter plot with average ± standard deviation. Two-way ANOVA with Dunnett's multiple comparisons test. F) Fold change for MDAMB231 cells during treatment up to 6 days with DMSO or imlunestrant (IML) at the indicated concentration. Growth curve with average ± standard deviation. Two-way ANOVA with Dunnett's multiple comparisons test.

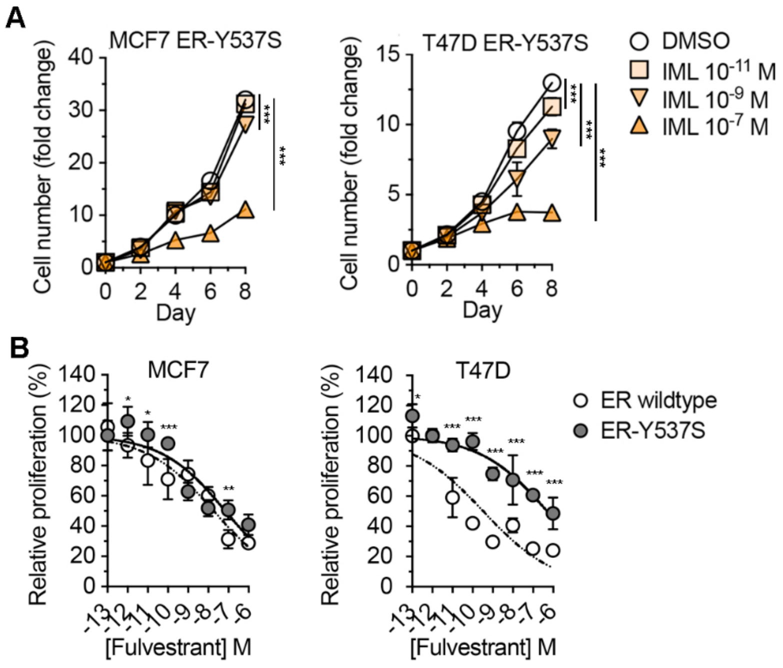

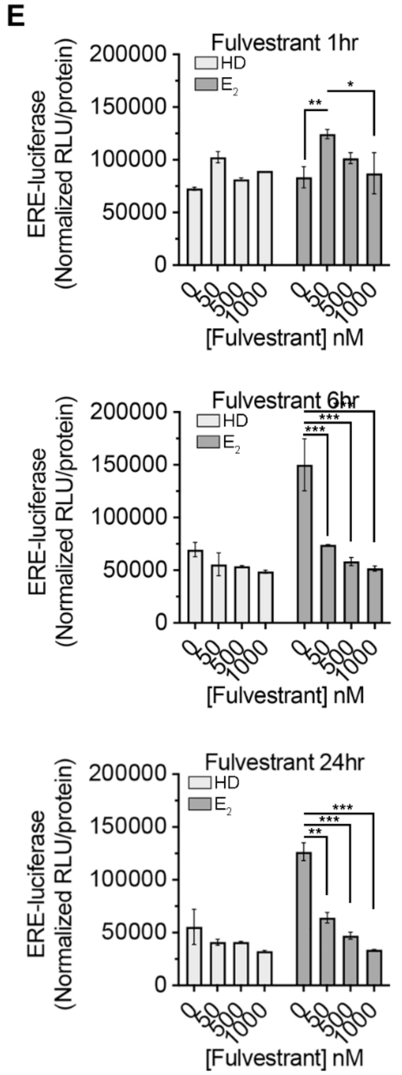

C

IC50 [nM]	MCF7		T47D	
	ER wildtype	ER-Y537S	ER wildtype	ER-Y537S
Imlunestrant	5.2	113	0.34	512
Fulvestrant	19	71	2.2	260

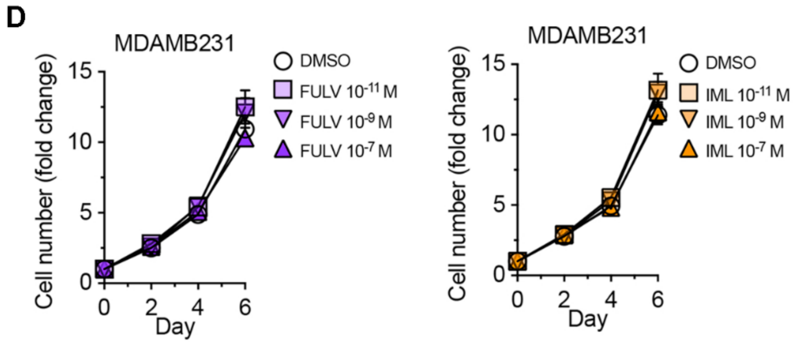

Supplemental Figure 2

A) Volcano plot of the differentially expressed genes following imlunestrant treatment in ER-Y537S expressing MCF7 or B) T47D cells. C) Volcano plot of the differentially expressed genes following fulvestrant treatment in ER-Y537S expressing MCF7 or D) T47D cells. Drug treatment for 12 hr. Dotted lines denote chosen cutoffs |log2FC| >= 1 and FDR < 10%. Orange indicates upregulated genes and blue indicates downregulated genes. ESR1 and estrogen-regulated genes are labeled with gene name when significant.

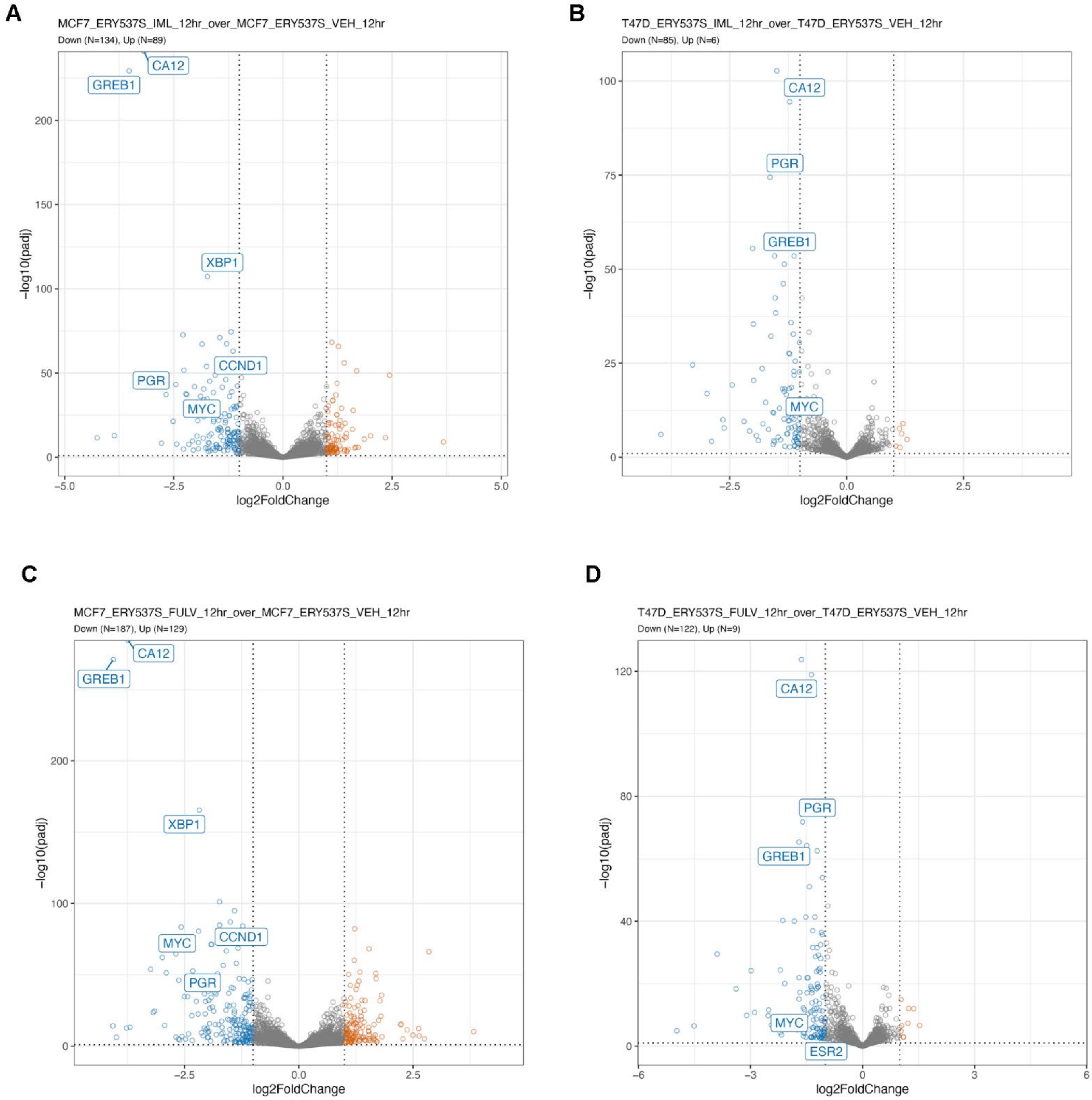

Supplement: Supplemental data [file jciinsight-10-188051-s008.pdf]
