# Supplementary figures and images for: Imlunestrant a next-generation oral SERD overcomes ESR1 mutant resistance in estrogen receptor–positive breast cancer
Source: JCI Insight. 2025 May 6;10(12):e188051. doi: 10.1172/jci.insight.188051 (PMC12226049; doi:10.1172/jci.insight.188051)

Figure 1F - ER

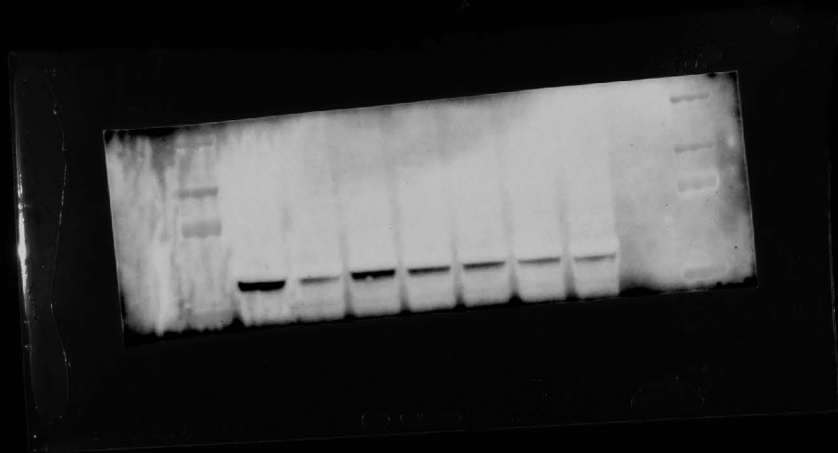

Figure 1F - GAPDH

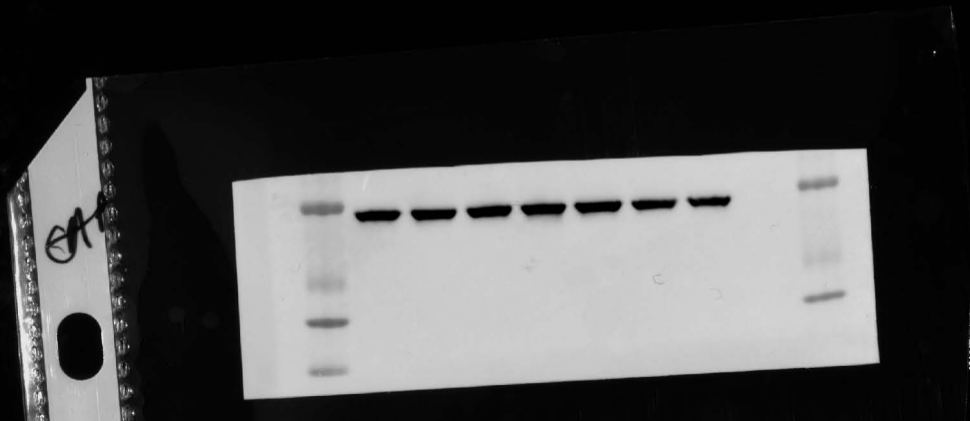

Figure 1G - ER

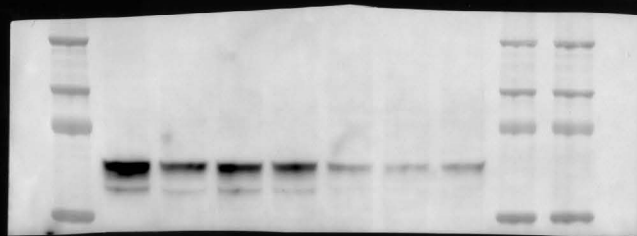

Figure 1G -  
GAPDH

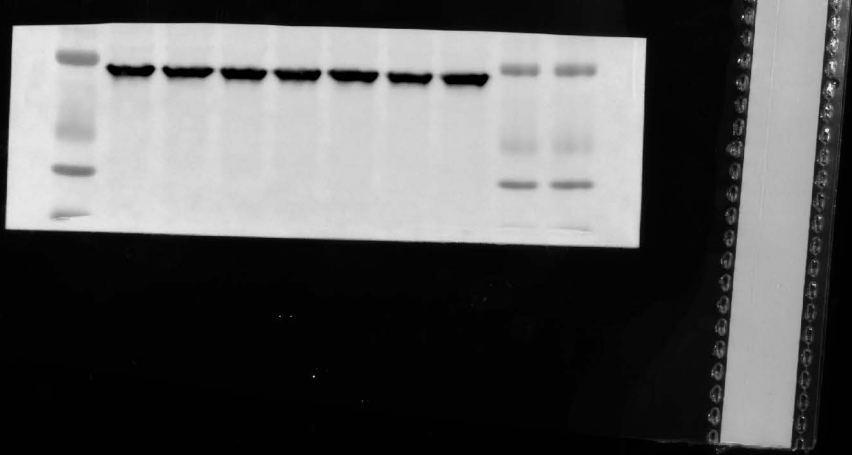

Supplement: Unedited blot and gel images [file jciinsight-10-188051-s009.pdf]
